# ADVANCING THE MEASUREMENT OF INTERGENERATIONAL CONTACT: BEYOND ATTITUDES TOWARD AGING

**DOI:** 10.1093/geroni/igz038.2334

**Published:** 2019-11-08

**Authors:** Shelbie Turner, Karen Hooker, Shannon E Jarrott

**Affiliations:** 1 Oregon State University, Corvallis, Oregon, United States; 2 Ohio State University, Columbus, Ohio, United States

## Abstract

In our presentation, we will offer insights into our process of creating and validating a comprehensive theory- and evidence- informed measure of intergenerational contact that expands beyond the measurement of age-related attitudes. While attitudinal shifts are an important construct related to intergenerational contact and its impact on ageism, efforts to “Reframe Aging” require a more nuanced understanding of the mechanisms by which intergenerational contact can have positive impacts on individuals, families, and communities. Intergenerational contact is dynamic; it varies both between- and within- people, dyads, and places, as well as over time. Our measure includes quantity and qualities of intergenerational contact, including the extent to which the contact is between family vs. non-family members. Unlike existing measures of intergenerational relationship, ours reflects young persons’ and older adults’ intergenerational relationships. A psychometrically valid instrument of intergenerational contact is an essential first-step for determining how aging can be reframed through intergenerational interactions.

